# Long-term probiotic intervention mitigates memory dysfunction through a novel H3K27me3-based mechanism in lead-exposed rats

**DOI:** 10.1038/s41398-020-0719-8

**Published:** 2020-01-22

**Authors:** Jie Xiao, Tian Wang, Yi Xu, Xiaozhen Gu, Danyang Li, Kang Niu, Tiandong Wang, Jing Zhao, Ruiqing Zhou, Hui-Li Wang

**Affiliations:** 1 Engineering Research Center of Bio-process, Ministry of Education, Hefei University of Technology, Hefei, China; 2 School of Food and Bioengineering, Hefei University of Technology, Hefei, China

**Keywords:** Molecular neuroscience, Psychiatric disorders, Epigenetics and behaviour

## Abstract

Chronic lead exposure is associated with the development of neurodegenerative diseases, characterized by the long-term memory decline. However, whether this pathogenesis could be prevented through adjusting gut microbiota is not yet understood. To address the issue, pregnant rats and their female offspring were treated with lead (125 ppm) or separately the extra probiotics (10^10^ organisms/rat/day) till adulthood. For results, memory dysfunction was alleviated by the treatment of multispecies probiotics. Meanwhile, the gut microbiota composition was partially normalized against lead-exposed rats, which in turn mediated the memory repairment via fecal transplantation trials. In the molecular aspect, the decreased H3K27me3 (trimethylation of histone H3 Lys 27) in the adult hippocampus was restored with probiotic intervention, an epigenetic event mediated by EZH2 (enhancer of zeste homolog 2) at early developmental stage. In a neural cellular model, EZH2 overexpression showed the similar rescue effect with probiotics, whereas its blockade led to the neural re-damages. Regarding the gut–brain inflammatory mediators, the disrupted IL-6 (interleukin 6) expression was resumed by probiotic treatment. Intraperitoneal injection of tocilizumab, an IL-6 receptor antagonist, upregulated the hippocampal EZH2 level and consequently alleviated the memory injuries. In conclusion, reshaping gut microbiota could mitigate memory dysfunction caused by chronic lead exposure, wherein the inflammation–hippocampal epigenetic pathway of IL-6-EZH2-H3K27me3, was first proposed to mediate the studied gut–brain communication. These findings provided insight with epigenetic mechanisms underlying a unique gut–brain interaction, shedding light on the safe and non-invasive treatment of neurodegenerative disorders with environmental etiology.

## Introduction

Alzheimer’s disease (AD) and Parkinson’s disease (PD) are among the most common neurodegenerative disorders characterized by progressive cognitive decline and memory loss^1,2^. Most AD cases arose from environmental and genetic risk factors, the interaction of which may perturb hippocampus-dependent learning and memory, accelerate cognitive decline, and contribute to the development of neurodegenerative diseases^1,3^. Of note, low-level environmental exposure to lead during early childhood was associated with the pathogenesis of AD and PD^4,5^.

Lead/Pb is a ubiquitous environmental toxicant that continues to threaten human health on a global scale^6,7^. Adverse psychiatric consequences caused by lead were characterized by deficits of learning and memory^8^. For the treatment of lead toxicity, chelation therapy was used to reduce blood lead levels (BLLs). However, this therapy was proven ineffective in coping with low-level exposures (BLL < 45 μg/dl) and failed to reverse the related memory deficits^9^. In light of this, alternative strategies of resolving lead-induced onset of neurodegenerative diseases are warranted to be developed, where gut microbiota (GM) intervention was assumed to be a competent candidate.

The human gastrointestinal tract is inhabited by 10^13^–10^14^ microorganisms, forming a complex community composed of >1000 species^10,11^. The community is dominated by the bacteria, with approximately 90% of species belonging to Firmicutes and Bacteroidetes^12^. Growing evidence indicates that GM can impact the brain (so called “microbiome–gut–brain axis”), whereas it communicates via hormonal, neuronal, or immune pathways^13–15^. More importantly, dysbiosis of GM contributes to the development of AD or other cognition-related psychiatric disorders^16,17^. To date, some attempts have been made to use probiotics to protect organisms from lead toxicity, in which, however, only acute lead poisoning and non-central nervous system (non-CNS) organs were involved in these studies^18,19^. Meanwhile, lead removal by *Lactobacillus* strains was primarily attributed to their physical contact, adsorption, and binding with lead ions^18^, leaving chronic exposure occasions, characterized by disparate exposure timing or routes, unresolved.

The precise mechanisms of GM in shaping brain functions are unclear, but multiple pathways were previously suggested, highlighted by inflammatory pathways^20^. In the molecular perspective, numerous molecules in the brain were implicated in GM-regulated neuronal activity, specified by neurotransmitters, their receptors, neuroplasticity-related proteins, neurotrophic factors, etc.^21,22^. However, epigenetic factors were rarely proposed. Epigenetics is defined as heritable changes in gene expression that occur without DNA sequence changes^23,24^. Epigenetic regulators can have dramatic consequences on the CNS^6,25^, and their perturbations were associated with a range of psychiatric disorders^26^. Interestingly, developmental lead exposure could lead to multiple epigenetic aberrations^27,28^. Specific to the individual epigenetic form, histone acetylation and methylation were profoundly implicated in lead neurotoxicity, as evidenced by our previous findings^29,30^. Among the various modifications, histone methylation at Lys 27 of histone H3 (H3K27me) is relatively stable and conventionally controls gene repression^31^. Although epigenetic roles in connecting GM to multiple host organs have been appreciated^32,33^, their implications in the microbiome–gut–brain axis remain elusive.

In the present study, memory dysfunction induced by low-level lead exposure was mitigated by probiotic administration. Accompanied by it, gut microbial composition was remodeled, and interleukin-6 (IL-6) and hippocampal H3K27me3 were implicated in the repair pathways. This investigation introduces microbiota-oriented strategy to treat neurodegenerative diseases, providing insight with novel roles of neural epigenetics in the studied gut–brain connections.

## Materials and methods

### Animals and study design

Sprague-Dawley (SD) rats were obtained from the Laboratory Animal Center of Anhui Medical University. All animal procedures were carried out in accordance with National Institute of Health Guide for the Care and Use of Laboratory Animals and were approved by the Institutional Animal Care and Use Committee of Hefei University of Technology, China. Three dams were used in each treatment group, and each dam could give birth to 10–14 offspring on average. The pups were randomly selected after weaning in each group and subjected to the subsequent trials. Lead acetate (125 ppm) was administered ad libitum in drinking water. Lead exposure toward pregnant dams began at 1 week after co-housing with the adult males and lasted postnatally toward female pups. The postnatal exposure was carried out through their mothers at developmental stage (till postnatal day (PND) 21) and then directly after weaning (PND 21) till sacrifice. The lead-exposed model was referenced to the previous studies^34–37^. For probiotic treatment, multispecies probiotics containing *Bifidobacterium longum* BL986, *Lactobacillus acidophilus* LA1063, *Lactobacillus fermentum* LF26, *Lactobacillus helveticus* LH43, *Lactobacillus paracasei* LPC12, *Lactobacillus rhamnosus* LRH10, and *Streptococcus thermophilus* ST30 (OsteoForm, Nanjing, Jiangsu, China) were supplied at a dosage of 10^10^ organisms/rat/day for 4 ml in sterilized water from in utero till sacrifice (the same period with lead exposure) in a specialized independent container (3 × 10^10^ organisms in 12 ml sterilized water for 3 rats in a cage, and the administration was normally completed in 2 min), to assure that treatment of lead and probiotics were spatiotemporally separated (Supplementary Video S1). The food consumed by each groups of rats (g/rat) was 13.89 ± 2.43 for ctrl, 11.06 ± 1.47 for pb, 9.2 ± 1.44 for pb+prob, and 11.44 ± 2.58 for prob. The water consumed by each groups of rats (g/rat) was 21.07 ± 3.06 for ctrl, 15.08 ± 2.76 for pb, 12.21 ± 2.39 for pb+prob, and 16.63 ± 4.86 for prob. Female rats were subjected to the subsequent experiments due that no uniformly significant memory damages or probiotic rescue (manifested by the crucial measures in behavioral test and spine morphology) were observed for male SD rats in the studied context (Supplementary Fig. S1).

Female rats were assigned randomly to one of the five groups (Supplementary Fig. S2). The experimental timelines are summarized as follows: Group a was subjected to morphological analysis and EZH2 immunoblots at PND 22; Group b was subjected to a behavioral test battery consisting of the Y-maze test at PND 55 and Morris Water Maze (MWM) test at PNDs 56–61. Following sacrifice at PND 68, the animals were subjected to morphological analysis and H3K27me3 immunoblots and immunostaining, as well as serum collection for lead concentration measurement, cytokine examination, and cellular experiments. The feces were collected prior to behavioral assessment for microbiome analysis; Group c was intraperitoneally injected with Tocilizumab (8 mg/kg, Roche Pharma, Utsunomiya, Tochigi, Japan) or normal saline at PND 14, and a week later, they were subjected to the EZH2 quantifications; Group d was injected with Tocilizumab or normal saline at PNDs 14, 28, and 42 and subsequently subjected to the behavioral test battery described in Group b; Group e was administered with feces from either lead or lead+probiotics treated 8-week-old rats for 5 weeks and then subjected to the behavioral test battery described in Group b. Specifically, microbiota was freshly harvested, and the fecal content was pooled, homogenized in a 1:4 ratio in sterile solution (1× phosphate-buffered saline: 80% glycerol, ratio 1:1), centrifuged at 800 rpm for 3 min, and the supernatant was collected and aliquoted. The fecal transplantation began at PND 21 of recipient rats (200 μl cocktail/rat/day) via gavage administration and lasted till PND 56.

Typical sample size was chosen in accordance with previous publications^38–40^ and is similar with those generally employed in the field. For animal studies, as calculated by power analysis^41^, the total sample size estimate was 13 (2 groups) based on the assumed effect size of 0.6, with the expected *α* and power values set as 0.05 and 0.8, respectively.

### Antibodies and reagents

Anti-H3K27me3 (#ab6002), anti-H3K4me3 (#ab8580), anti-H3K4me2 (#ab32356), and anti-H3 antibodies (#ab1791) were purchased from Abcam China (Shanghai, China). Anti-EZH2 (#07-689) antibody was purchased from EMD Millipore (Shanghai, China). Anti-β-actin (#4970S) was purchased from Cell Signaling Technology (Shanghai, China). DAPI (4,6-diamidino-2-phenylindole; #C0060) was purchased from Solarbio (Beijing, China). All other reagents were of the highest analytical grade.

### Behavioral assessment

Behavioral measures to assess spatial memory began when the offspring reached adulthood. All behavioral experiments were video-recorded and automatically scored by the Smart tracking software (ANY-maze; Stoelting, Shanghai, China). After sample collection at PND 68, brain lead level and BLL were determined using the double-channel atomic fluorescence spectrometer (TITAN Instruments, Beijing, China), according to the standard protocol of State Standard of the People’s Republic of China. Analysis of behavioral outcome was performed by a third person without being informed of actual grouping.

MWM test was conducted according to our previous study^35^. Briefly, experiments were performed in a circular pool with a diameter of 1600 mm and depth of 700 mm, which was filled with water to a depth of 400 mm. The temperature was maintained at 25 °C. During training days, each rat got four trials daily for 5 consecutive days to find the hidden platform. When it reached the platform, it was allowed to stay on it for 30 s. If it failed to touch the platform within 90 s, it would be guided to stay there for 30 s. The rats that kept running alongside the pool wall and failed to look for the platform on the first day were excluded from the subsequent trials, with the criteria previously established^42^. The platform was removed on the sixth day, the test day, and the randomly selected rats were subjected to a 90-s trial to reflect its ability of memory retention. The rats were measured in an alternating fashion. The moving tracks were video-recorded (Supplementary Videos S2 and S3) and automatically scored by the Smart tracking software (ANY-maze; Stoelting, Shanghai, China). The platform-crossing times, time spent on the target quadrant, and total moving distances were analyzed.

Y-maze test was performed as follows: at the beginning of each experimental session, each rat was placed on the central platform. The rat was then allowed to explore all three arms of the maze and the number of spontaneous alternations (defined as number of successive triplet entry into each of the three arms without any repeated entries) was monitored in a 10-min test session. The rats were measured in an alternating fashion. The percentage of spontaneous alternation was calculated as the ratio of “the number of alternations” to “total number of arm entries − 2”. The alternation percentage was used as a parameter for the working memory-related behavior.

### Spine counting

Hippocampus was removed from the decapitated rats within 1 min at PNDs 22 and 68, and then subjected to Golgi–Cox staining for spine density analysis with respect to CA1 and dentate gyrus (DG) regions, as described previously^43^. Approximately 50 granule neurons and pyramidal neurons in the hippocampal DG and CA1 regions were imaged with a Nikon microscope (Eclipse 80i, Nikon, Tokyo, Japan) using a ×20 objective lens; specifically, the dendritic sections were imaged with a ×100 objective lens. The spines were selected from dendrites with secondary and tertiary branching. Approximately 50 granule or pyramidal cells were randomly chosen from the intact cells occurring in a microscopic field, and all spines located in the secondary and tertiary branching were counted and the mean spine number per cell was calculated. The damaged or illegible cells were eliminated, including the truncated ones by slicing, severely damaged ones by Cox staining, and the legible ones <10 μm. The selection and statistics were carried out using a double-blind strategy. Spine density (spine number per 10 μm dendrite) was calculated by using the MATLAB software.

### Cell culture

PC-12 cells (undifferentiated and differentiated lines) were purchased from cell repository of Chinese Academy of Sciences and previously authenticated by DNA Fingerprinting test and tested for mycoplasma contamination. The cell line was cultured at 37 °C, 5% CO_2_ in humidified atmosphere with RPMI1640 medium supplemented with 5% fetal bovine serum (FBS), 10% horse serum, and 1% penicillin–streptomycin. To initiate growth, PC-12 cells were plated onto six-well medium coated with poly-d-lysine/lamine. Considering Pb exposure, lead acetate (5 μM) was added at 24 h of growth. Undifferentiated cell lines were re-inoculated with fresh media supplemented with nerve growth factor (50 ng/ml) at 24 h. Following additional incubation for 72 h (with medium changed every 36 h), morphological analysis was performed according to images from fluorescence microscopy (Nikon, Tokyo, Japan). OE-EZH2, KD-EZH2, or empty (green fluorescent protein) plasmids (500 ng), which were constructed in our previous investigations^30^, were transfected into cells using Lipofectamine 3000 (Thermo Fisher Scientific, Beijing, China) at 24 h of growth. To establish a cellular model impacted by GM, the growth medium was supplemented with 40 μl of sterilized sera from healthy rats treated with probiotics or placebo as the growth began^44^.

### Microbiome analysis

The fresh feces from adult female rats (PND 68) were collected, homogenized, and subjected to DNA extraction using the HiPure Stool DNA Kit B (Magen, Guangzhou, Guangdong, China), according to the manufacturer’s instructions. Subsequently, 1 ng/μl of DNA was subjected to 16SV4 rRNA amplification. The primer pair used in V4 amplification is 515F (5′-GTGYCAGCMGCCGCGGTAA-3′) and 806R (5’-GGACTACNVGGGTWTCTAA-3′). The PCR products were extracted and purified using the GeneJET Gel Extraction Kit (Thermo Fisher Scientific, Beijing, China), according to the manufacturer’s instructions.

Library was constructed using the Ion Plus Fragment Library Kit (Thermo Fisher Scientific, Beijing, China). Following the Qubit quantification, the pooled amplicons were subjected to IonS5^TM^XL for sequencing. Data analysis was conducted with the following steps: (1) data split: single-end reads were assigned to samples based on their unique barcode and truncated by cutting off the barcode and primer sequence; (2) data filtration: quality filtering on the raw reads were performed under specific filtering conditions to obtain the high-quality clean reads according to the Cutadapt quality controlled process; (3) chimera removal: the reads were compared with the reference database (Silva database, https://www.arb-silva.de/) using UCHIME algorithm to detect chimera sequences, and then the chimera sequences were removed; (4) operational taxonomic unit (OTU) production: sequences analysis were performed by the Uparse software (Uparse v7.0.1001, http://drive5.com/uparse/). Sequences with ≥97% similarity were assigned to the same OTUs. Representative sequence for each OTU was screened for further annotation; (5) species annotation: for each representative sequence, the Silva Database (https://www.arb-silva.de/) was used based on Mothur algorithm to annotate taxonomic information; (6) phylogenetic relationship construction: multiple sequence alignment was conducted using the MUSCLE software (Version 3.8.3, http://www.drive5.com/muscle/); (7) data normalization: OTU abundance information was normalized using a standard of sequence number corresponding to the sample with the least sequences.

Differences in microbial communities between groups were investigated through the phylogeny-based unweighted UniFrac distance metrics. Alpha diversity and UPGMA clustering were performed with the respective QIIME scripts (Version 1.9.1). R software (Version 2.15.3) was used to depict the non-metric multi-dimensional scaling (NNMDS) graph and analyze the inter-group differences of beta diversity. Linear discriminant analysis (LDA) effect size (LEfSe) analysis was performed using the LEfSe software according to the manufacturer’s instructions, with the default filter value of LDA score set as 4.0.1. The datasets generated during microbiome analysis are available in the Mendeley repository, 10.17632/n384ds7xn4.1.

### Immunoblots and immunostaining

Hippocampi of female rats were homogenized in 200 μl ice-cold lysis buffer containing a cocktail of protein phosphatase and protease inhibitors. Lysates were then centrifuged for 7 min at 14,000 × *g* at 4 °C. Supernatants were collected in new Eppendorf tubes and quantified using the BCA Protein Assay Kit (Beyotime, Shanghai, China). The protein supernatant (100 μg) was boiled for 10 min and mixed with equal amount of 2× sodium dodecyl sulfate (SDS) loading buffer. Equal amount of proteins (25 μg) were loaded on 10% SDS-polyacrylamide gel electrophoresis gel for electrophoresis and then transferred to a polyvinylidene difluoride (PVDF) membrane (Millipore, MA, USA). After being blocked for 1 h by 5% skim milk at room temperature, the PVDF membrane was cut into strips according to molecular weight of EZH2 (98 kD), H3K27me3 (15 kD), and β-actin (42 kD). Subsequently, the strips were incubated with the primary and secondary antibodies sequentially and developed using the enhanced chemiluminescence immunoblotting detection system (Thermo, Beijing, China). The anti-EZH2 antibody (Millipore, Shanghai, China, 1:1000) and anti-H3K27me3 antibody (Abcam, Shanghai, China, 1:1000) were used to measure the EZH2 and H3K27me3 levels at PNDs 22 and 68, respectively. Densitometry was assessed with the Image J software, with protein quantity normalized to H3 or β-actin.

After >24 h of fixation in 4% polyformaldehyde, brains were wrapped in Tissue-Tek O.C.T. Compound (Radnor, PA, USA) and frozen to −20 °C immediately. The fixed tissues were sectioned coronally at a width of 40 μm with a microtome cryostat. A one-in-six section from bregma −2.92 mm to bregma −4.29 mm was subjected to immunostaining. In all, 0.2% of Triton X-100 was added to make the tissue slices permeable. After being blocked by 5% FBS, the slices were sequentially incubated in rabbit anti-histone H3 (tri methyl K27) monoclonal antibody (Abcam, Shanghai, China; 1/200) overnight and goat anti-rabbit IgG (H+L) secondary antibody (Boster, Wuhan, Hubei, China; 1/50) for 1 h at 4 °C. Nuclei were stained using a goat polyclonal DAPI (Solarbio, Shanghai, China; 1/5000). Fluorescent imaging was performed at a ×20 objective using Nikon fluorescence microscope.

### Enzyme-linked immunosorbent (ELISA) assay

Levels of plasma cytokines (including IL-6, IL-10, tumor necrosis factor-α, interferon-γ, and granulocyte colony-stimulating factor (G-CSF)) were all measured using ELISA kits (mlbio, Wuhan, Hubei, China), according to the manufacturer’s instructions. Briefly, 10 μl of blood serum in adult rats (PND 68) with lead and/or probiotic treatment was collected and subjected to ELISA analysis.

### Statistical analysis

Graph data are presented as mean ± SEM. Statistical analysis was performed using the SPSS software. When the experimental setting contained four groups (ctrl, pb, pb+prob, prob), two-way analysis of variance (ANOVA) was used to perform single endpoint analysis. When the experiment was designed as three groups (ctrl, pb, pb+prob), one-way ANOVA was used to perform single endpoint analysis. When time factor was incorporated into the statistics (MWM test with time variables), repeated-measures ANOVA was performed with Turkey post hoc multiple comparisons (main treatment effect). Unpaired, two-tailed *t* test was used to perform two-group comparisons and was false discovery rate (FDR) corrected when multiple comparisons (>3) were performed. Statistical tests were chosen appropriate to sample sizes, variable factors, or group number for comparisons. The homogeneity of variances was assessed with Levene test. In case of a non-parametric distribution of data, Mann–Whitney *U* test was used. NMDS analysis was performed, centered, and scaled to unit variance (R function prcomp). Permanova was performed to make multiple comparisons of inter-group community structure. The data meet the assumptions for the specific statistical test we chose, and the tests were automatically adjusted when variances among groups differed. The number of samples examined in each analysis was shown in the legends and all data were obtained from at least triplicate repeats, and the experiments shown are replicated at least twice in the laboratory. Probability values of *p* ≤ 0.05 were regarded as statistically significant.

## Results

### Probiotics alleviated the lead-led memory impairment

**Fig. 1:**
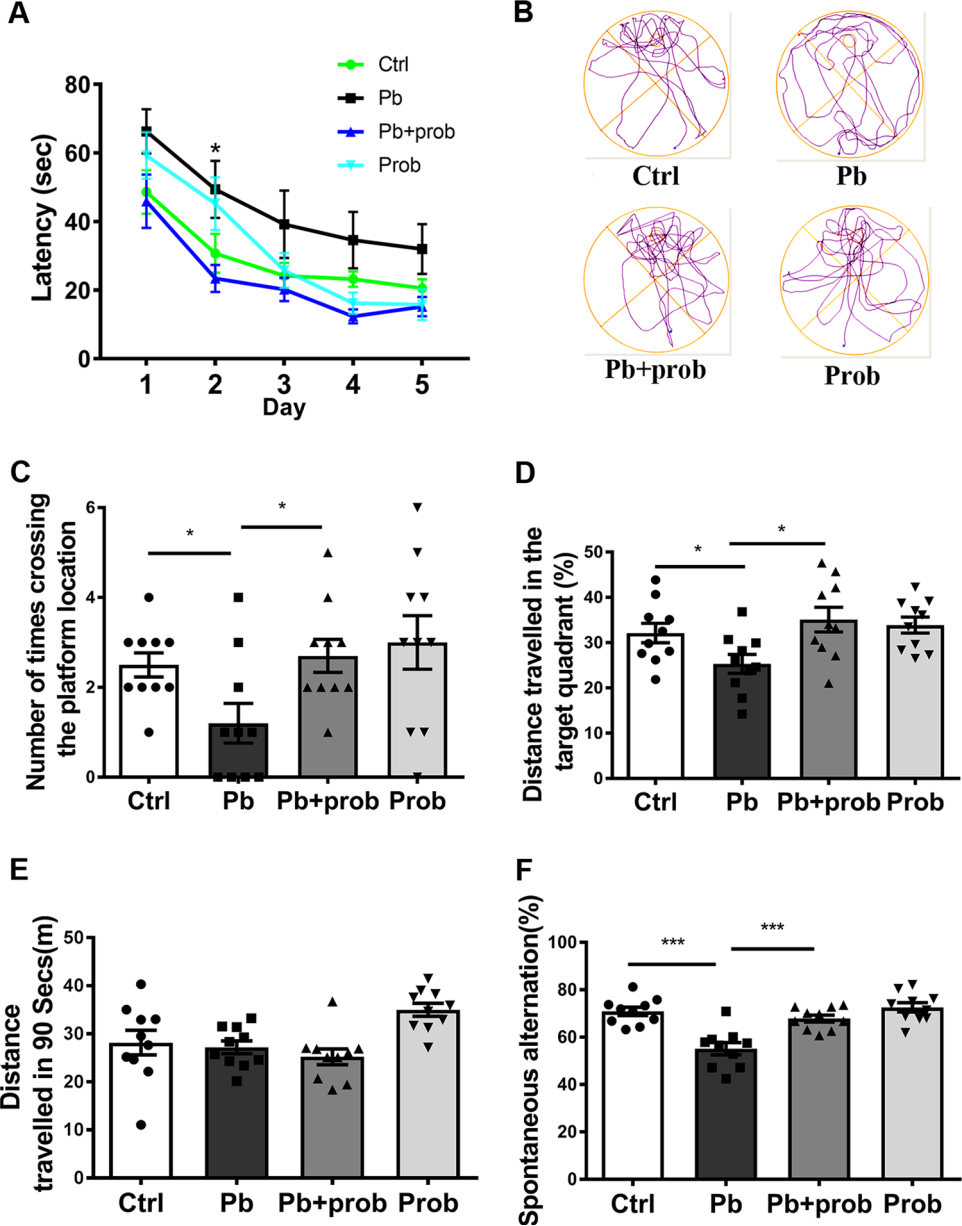
Probiotics alleviated the lead-led memory impairment. **a**–**e** MWM tests assessing capacities of rats to be trained to find the hidden platform (*n* = 10). Latency of rats to find the platform was recorded and analyzed during the training days (**a**). On the test day, based on their respective moving tracks (**b**), number of times crossing the hidden platform (**c**) and distance traveled in the target quadrant (**d**) were counted at PND 61. The total distance traveled was also recorded to evaluate the locomotor movement of rats (**e**). **f** Y-maze test assessing capacities of rats to perform the spontaneous alteration (*n* = 10). Spontaneous alteration percentages of each group, as well as their respective moving tracks are shown. Ctrl non-treated rats, Pb lead-treated rats, Pb+prob lead and probiotics-treated rats, prob probiotics-treated rats. The data are represented as mean ± SEM; ***p < 0.001, *p < 0.05. For **a**, repeated-measures ANOVA was performed and asterisk (*) refers to the significance of differences between the Pb and Ctrl groups on the indicated training day.

MWM test is a standard paradigm to assess the spatial memory of animals^45^. It was used to evaluate the effect of multispecies probiotics on the memory impairment caused by chronic lead exposure. As evidenced in Fig. 1a, during the training sessions, the lead-treated SD rats showed a prolonged latency to find the target platform (*p* = 0.0082 vs. ctrl), an observation rescued by the probiotic supplement (*p* < 0.001 for pb+prob vs. pb). Repeated-measures ANOVA showed that both the training days (*F* (3.092, 114.4) = 222.62, *p* < 0.001) and treatment (*F* (3, 37) = 11.67, *p* < 0.001) had a significant effect on the latency performance, and their interaction was insignificant (*F* (12, 148) = 0.5287, *p* > 0.05). In terms of performances on the test day, rats exposed by pb+probiotics crossed the zone of removed platform more times than the pb-treated rat in the given period (*p* < 0.05, Fig. 1b, c). Two-way ANOVA revealed a significant effect of probiotics on the outcome (*F* (1, 36) = 5.279, *p* = 0.0275). In parallel, with the addition of probiotics, the moving tracks of rats showed a biased distribution into the target quadrant than the pb group (*p* < 0.05, Fig. 1b, d). Two-way ANOVA revealed a significant effect of probiotics on the outcome (*F* (1, 36) = 6.777, *p* = 0.0133). These results indicated that the lead-led impairment of spatial memory could be reversed by probiotic administration. Meanwhile, the total distance traveled among groups did not show significant differences (Fig. 1e), suggesting that the moving alteration caused by probiotics is not related to the enhanced total movement. Besides, no significant variations of body weights were observed among rat groups during the tested durations (Supplementary Fig. S3).

In addition to MWM test, Y-maze is generally used to evaluate the spatial working memory. In Y-maze test, the percentage of alterations was indicative of valid working memories. According to the results (Fig. 1f), while chronic lead exposure considerably decreased the alteration rate of rats placed in maze (*p* < 0.001 vs. ctrl), the damage could be effectively alleviated by probiotic supplement (*p* < 0.001 vs. pb). Two-way ANOVA revealed that both lead (*F* (1, 36) = 26.55, *p* < 0.0001) and probiotics (*F* (1, 36) = 13.22, *p* = 0.0009) had significant effects on the outcome, and their interaction was significant (*F* (1, 36) = 7.717, *p* = 0.0086). The Y-maze test consolidates the beneficial effect of probiotic formulation on the lead-led memory impairment.

### Probiotics rescued the morphological abnormalities of dendritic spines

**Fig. 2:**
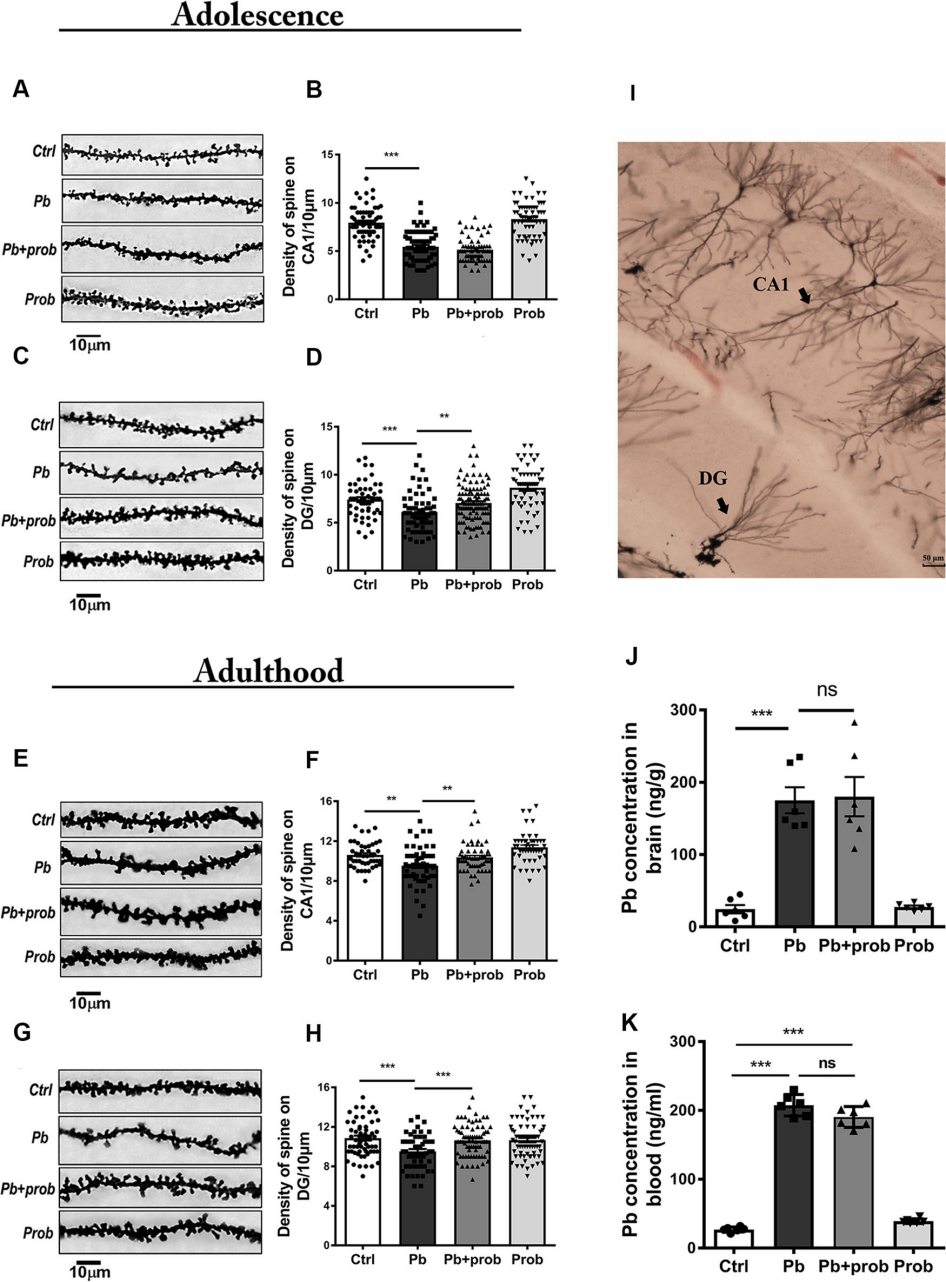
Probiotics rescued the morphological abnormalities of dendritic spines. At adolescence (PND 22), representative images (~50 cells, *n* = 6) and quantification of spine densities are shown in the region of CA1 (**a**, **b**) and DG (**c**, **d**). Each point in the graph represents a single cell subjected to analysis. At adulthood (PND 68), representative images and quantification of spine densities are shown in the region of CA1 (**e**, **f**) and DG (**g**, **h**). To manifest the tested hippocampal regions, typical morphologies of neurons in the regions of CA1 and DG are presented in the overall perspective (**i**). The scale bar represents 50 μm. **j**, **k** Lead concentration in the rat brains (**j**) and blood (**k**) of each group (*n* = 6). Ctrl non-treated rats, Pb lead-treated rats, Pb+prob lead and probiotics-treated rats, prob probiotics-treated rats. The data are represented as mean ± SEM; ***p < 0.001, **p < 0.01, NS *p* > 0.05.

It is known that dendritic spines are the morphological and structural basis for synaptic plasticity, learning, and memory^46^. To further confirm probiotic roles in rescuing the injured memories, Golgi–Cox staining was performed to describe hippocampal dendritic morphologies. It was observed from Fig. 2a–h that lead exposure resulted in the loss of dendritic spines in both CA1 (*p* < 0.001 for PND 22; *p* < 0.01 for PND 68 vs. ctrl) and DG regions (*p* < 0.001 vs. ctrl), irrespective of the developmental stages (PND 22 or PND 68). In response to microbial treatment, the spine densities were restored to a variable degree in adolescence or adulthood (*p* < 0.01 for DG at PND 22; *p* < 0.01 for CA1 at PND 68; *p* < 0.001 for DG at PND 68 vs. pb group, Fig. 2c–h), except for the CA1 region at PND 22 (*p* > 0.05 vs. pb group, Fig. 2b). Two-way ANOVA only revealed one significant interaction between lead and probiotic treatment in DG region at PND 68 (*F* (1, 248) = 8.585, *p* = 0.0037), where both lead (*F* (1, 248) = 9.086, *p* = 0.0028) and probiotics (*F* (1, 248) = 4.040, *p* = 0.0455) had main effects on the spine densities. Besides, the dendritic branching in the regions of CA1 and DG was also stained and manifested on a larger scale (Fig. 2i). The data here indicated that probiotics could rescue the lead-led loss of hippocampal spines both in adolescence and adulthood, with the effect of longer-term intervention more robust.

Given a couple of recent findings, some specific microbial strains could counteract the lead poisoning through direct adsorption and binding^18,19^. To investigate whether dynamic interaction occurs in the studied intervention process, brain lead levels were accordingly determined. Based on the results, chronic lead exposure elevated the average brain blood level from ~50 ng/g to >150 ng/g (*p* < 0.001, Fig. 2j), demonstrating the efficient penetration of blood–brain barrier. In addition, probiotic treatment did not yield any significant differences with pb-treated rats in both brain lead levels (*p* > 0.05, Fig. 2j) and BLLs (*p* > 0.05, Fig. 2k). No significant effect of probiotics on brain lead levels (*F* (1, 20) = 0.05924, *p* = 0.8102) or BLLs (*F* (1, 20) = 0.2945, *p* = 0.5934) was identified. Therefore, probiotics did not interfere with lead distribution in the condition of chronic exposure. As the joint effect of toxic/nutrient substances could take place in either exposure/kinetics or dynamics/effect phases^47^, it makes sense that the probiotic antagonism should be mainly ascribed to the superposed effect upon the intoxicating pathway of lead.

### Probiotic treatment restored specific microbiota changes in lead-exposed rats

In order to figure out the microbial compositions involved in the probiotic intervention, 16S rRNA sequencing was carried out using the IonS5^TM^XL sequencing platform. IonS5^TM^XL sequencing of fecal microbiota resulted in a total 1.3 million sequenced reads, corresponding to an averaged 84,565 reads per sample. After chimera removal, reads per sample were rarefied to 80,101. Based on the sequencing and data analysis, total 1554 OTUs were obtained, with 180 genera of microorganisms identified.

**Fig. 3:**
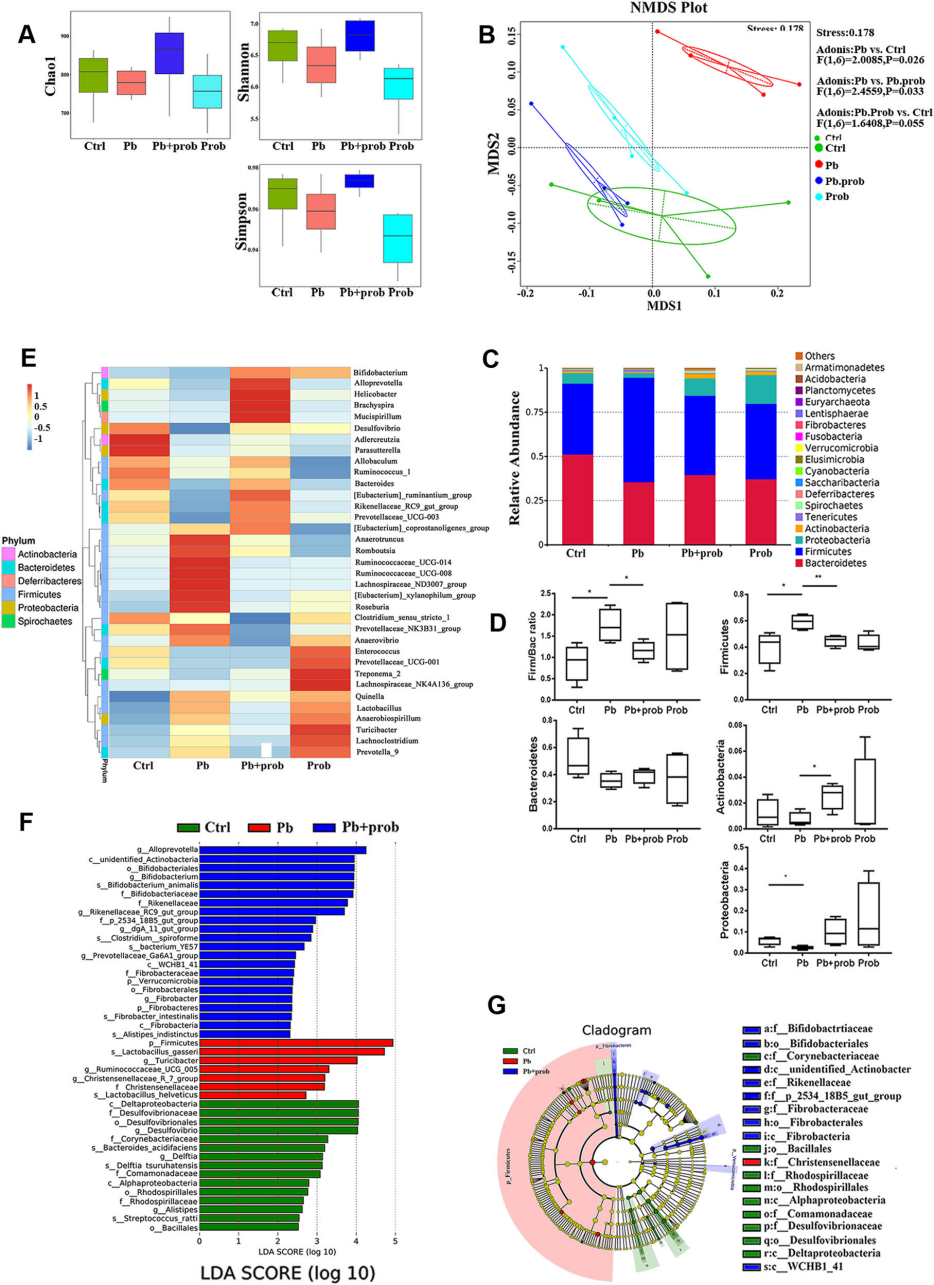
Probiotic treatment restored specific microbiota changes in lead-exposed rats (*n* = 6). **a** Analysis of alpha diversity-predicted diversity of gut microbiota by Chao1, Shannon, and Simpson analysis. **b** Non-metric multi-dimensional scaling (NMDS) analysis based on unweighted UniFrac metrics of gut microbiota where samples of rats from different groups are highlighted with different colors. The position and distance of data points indicated the degree of similarity in terms of bacterial taxonomies. The Permanova/Adonis analysis was shown on the right-upper corner. **c** Stacked bar chart shows microbiota composition at the phylum level. **d** Relative abundance of the major phyla of gut microbiota upon each treatment. **e** Heatmap shows relative abundance of representative microbiota at the genus level in four groups. **f** Microbial taxa identified to significantly differ in abundance (LDA score >2.0, *P* < 0.05) across the tested groups (Ctrl, Pb, Pb+prob) using LEfSe analysis. **g** A cladogram representation of LEfSe analysis. The plot shows microbial taxa from phylum to genus level, with taxa significantly enriched (*P* < 0.05) in the Pb group indicated in red and in Pb+prob indicated in blue. Abbreviations for microbial names are listed right to the graph. Ctrl non-treated rats, Pb lead-treated rats, Pb+prob lead and probiotics-treated rats, prob probiotics-treated rats. The data are represented as mean ± SEM; **p < 0.01, *p < 0.05. *p* Value was FDR corrected in multiple (>3) comparisons.

Compared to the lead group, microbial richness was in a reversed tendency by averages, from Chao1 analysis, as a consequence of probiotic administration (Fig. 3a). And in terms of phylogenetic diversity evidenced by Shannon and Simpson analysis, samples from the lead+probiotics group showed an increased diversity tendency by averages than lead-treated rats (Fig. 3a). In consequence, microbial richness and evenness tends to be resumed through probiotic administration.

NMDS is a rectified method of principal component analysis (PCA) to indicate the β-diversity among different microbial communities. Based on it, lead-treated rats displayed a dramatically distinct microbial composition from the control group (Permanova/Adonis, *F* (1, 10) = 2.8807, *p* = 0.004). Upon probiotic supplement, however, clustering of samples was located distant from the lead-treated samples and migrated to a similar profile with the healthy rats (Permanova/Adonis, *F* (1, 10) = 3.1441, *p* = 0.001 for pb vs. pb+prob; *F* (1, 10) = 1.3827, *p* = 0.062 for pb+prob vs. ctrl, Fig. 3b). Overall, it indicates that probiotic administration restores, to an extent, the gut microbial diversity disrupted by chronic lead exposure.

Concerning relative abundance at the phylum level, bacterial structure was altered by lead exposure, which was further remodeled by probiotic treatment (Fig. 3c). Specifically, Firmicutes (F) and Bacteroidetes (B) occupied the most abundant phyla in each group, and relative proportions of both taxa differed upon distinct treatment. F/B ratio was aberrantly increased by the lead treatment and then partially restored by the probiotic supplement (FDR < 0.05 for lead vs. ctrl, FDR < 0.05 for lead+probiotics vs. lead, Fig. 3c, d). With respect to Proteobacteria, lead exposure reduced its average abundance from 0.059 to 0.026 (FDR < 0.05), an alteration reversed by the probiotics to a level of 0.098. The restoration of microbial composition was also characterized by the case of Actinobacteria (average 0.007 vs. 0.012 for lead vs. ctrl; average 0.025 vs. 0.007 for lead+probiotics vs. lead, FDR < 0.05, Fig. 3c, d). On the basis of findings here, the disrupted microbial compositions were re-normalized by probiotic intervention.

Subsequently, the relative abundance of specific bacteria was manifested at the genus level (Fig. 3e). Probiotic treatment led to higher proportions of *Helicobacter*, *Bifidobacterium*, and *Bacteroides*, as well as concomitant lower proportions of *Anaerovibrio*, *Ruminococcaceae_UCG-008*, and *Lactobacillus*. The abundance of *Enterococcus* remained invariable upon probiotic treatment. Interestingly, probiotic supplement led to divergent alterations of *Bifidobacterium* and *Lactobacillus* species, indicating that probiotics might exert beneficial effect through optimization of microbiota architecture.

LDA was then used to analyze the microbial taxa contributing to diversity differences between the groups. Using the cutoff value of 2.0, it was found that seven taxa were significantly enriched in the lead-treated group, including the phylum Firmicutes and species *Lactobacillus gasseri*/*helveticus* (FDR < 0.001, compared with the ctrl and pb+prob groups, Fig. 3f, g). Following probiotic treatment, a couple of phyla like Verrucomicrobia and Fibrobacteres, as well as the order Bifidobacteriales, were enriched compared with the ctrl and pb groups (FDR < 0.001, Fig. 3f, g).

### Reshaping GM repaired the injured memory

**Fig. 4:**
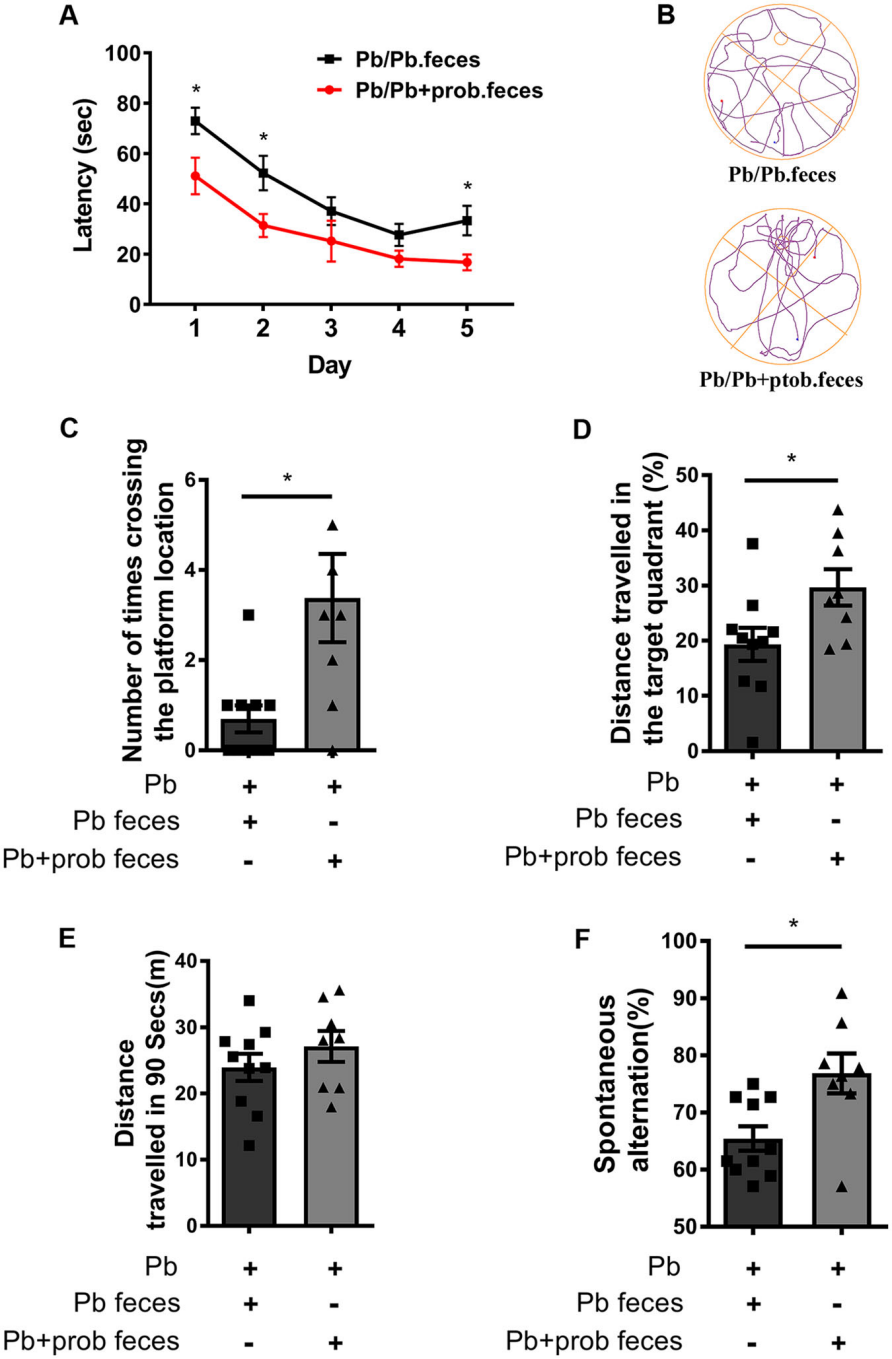
Fecal transplantation alleviated the lead-led memory impairment. **a**–**e** MWM tests assessing the capacities of rats to be trained to find the hidden platform, and the comparisons were performed between recipient rats transplanted with feces from either lead (*n* = 10) or lead+probiotics (*n* = 8) treated donors. Latency of rats to find the platform was recorded and analyzed during the training days (**a**). On the test day, based on their respective moving tracks (**b**), number of times crossing the hidden platform (**c**) and distance traveled in the target quadrant (**d**) were counted at PND 61. The total distance traveled was also recorded to evaluate the locomotor movement of rats (**e**). **f** Y-maze test assessing the capacities of rats to perform the spontaneous alteration, and the comparison was performed between recipient rats transplanted with feces from either lead (*n* = 10) or lead+probiotics (*n* = 8) treated donors. Spontaneous alteration percentages of each group were shown. Pb lead-treated rats, Pb.feces fecal transplantation from donor rats treated with lead, Pb+prob.feces fecal transplantation from donor rats treated with lead+probiotics. The data are represented as mean ± SEM; *p < 0.05. For **a**, repeated-measures ANOVA was performed and asterisk (*) refers to the significance of differences between the Pb/Pb.feces and Pb/Pb+prob.feces groups on the indicated training day.

In order to investigate whether the remodeled GM mediated the probiotic intervention against memory impairment, we further administered recipient rats with feces derived from donors with the treatment of lead or lead+prob, respectively. The donors were supposed to contain the disrupted (disease donor) or remodeled microbiota (intervention donor) as shown in Fig. 3. As evidenced in Fig. 4a, during the training sessions, the rat latency to find the target platform was shortened via the transplantation of feces from intervention donors (*p* = 0.0405). In terms of performances on the test day, the rats administered with feces from intervention donors crossed the zone of removed platform more times than the control recipient rats in the given period (*p* = 0.0112, Fig. 4b, c). In parallel, with microbiome reshaped by intervention donor feces, the moving tracks of lead-exposed rats showed a biased distribution into the target quadrant (*p* = 0.0342, Fig. 4b, d). Meanwhile, the total distance traveled among groups did not show significant differences (Fig. 4e). Besides, the Y-maze test showed that the rats transplanted with feces from intervention donors performed better in the spontaneous alternation (*p* = 0.013, Fig. 4f), validating the results of MWM test. In summary, these results demonstrated that probiotics improved the injured memory via reshaping the gut microbiome.

### Probiotics reversed the H3K27me3 level in the rat hippocampus

**Fig. 5:**
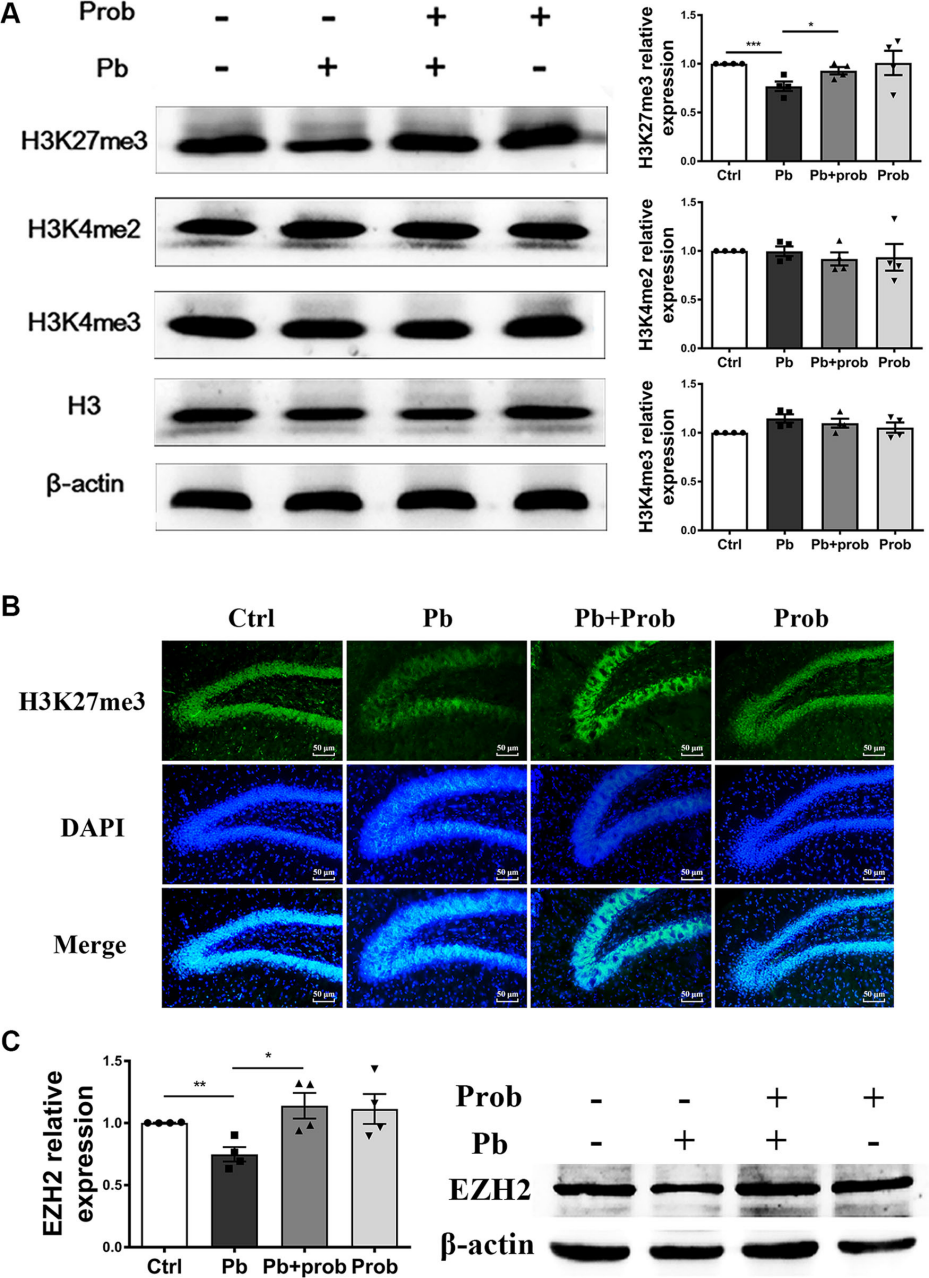
Probiotics reversed the H3K27me3 level in the rat hippocampus (*n* = 4). **a** Immunoblots and quantification of H3K27me3, H3K4me2, and H3K4me3 levels in the hippocampus of adult rats. **b** Immunostaining of H3K27me3 (green) and nucleus (blue) in DG region of adult rats hippocampus. The scale bar represents 50 μm. **c** Immunoblots and quantification of EZH2 levels in the hippocampus of adolescent rats. Ctrl non-treated rats, Pb lead-treated rats, Pb+prob lead and probiotics-treated rats, prob probiotics-treated rats. The data are represented as mean ± SEM; ***p < 0.001, **p < 0.01, *p < 0.05.

Given our previous findings that lead exposure elicited changes of histone methylations in the rat hippocampus (data in press), epigenetic regulation is hypothesized to lie at the central position of the studied gut–brain communication. To test this hypothesis, various histone methylations in the rat hippocampus were measured in response to probiotic supplement. Contrary to H3K4me2 and H3K4me3, the disrupted presence of H3K27me3 was significantly restored with probiotics at PND 60 (*p* < 0.05 vs. pb group), suggesting that hippocampal H3K27me3 levels might be impacted by probiotic administration and the consequent microbial orientation (Fig. 5a). Two-way ANOVA revealed no interaction of lead and probiotic treatment on the H3K27me3 levels (*F* (1, 12) = 1.164, *p* = 0.3019). Furthermore, immunostaining gave a visible picture of the presence of H3K27me3 in situ, which consisted of alterations detected by immunoblotting analysis (Fig. 5b).

H3K27me3 is introduced by PRC2 complex consisting of EZH2, EED, Suz12, and RbAp48^48^. To identify the prime candidates leading to a restored H3K27me3 level, the abundance of EZH2 protein was examined both at PNDs 21 and 60. Consistent with the previous study^49^, EZH2 attenuated rapidly as development progressed, and no apparent proteins could be detected in any samples at PND 60 (data not shown). Data from PND 21 revealed, however, that probiotics significantly elevated the EZH2 level in the rat hippocampus (*p* < 0.05 vs. pb group, Fig. 5c). Two-way ANOVA revealed a significant effect of probiotics on the EZH2 levels (*F* (1, 12) = 8.887, *p* = 0.0115). These results reflected a coordinated relation between EZH2 and H3K27me3.

### EZH2-H3K27me3 mediated the probiotic alleviation of lead neurotoxicity

**Fig. 6:**
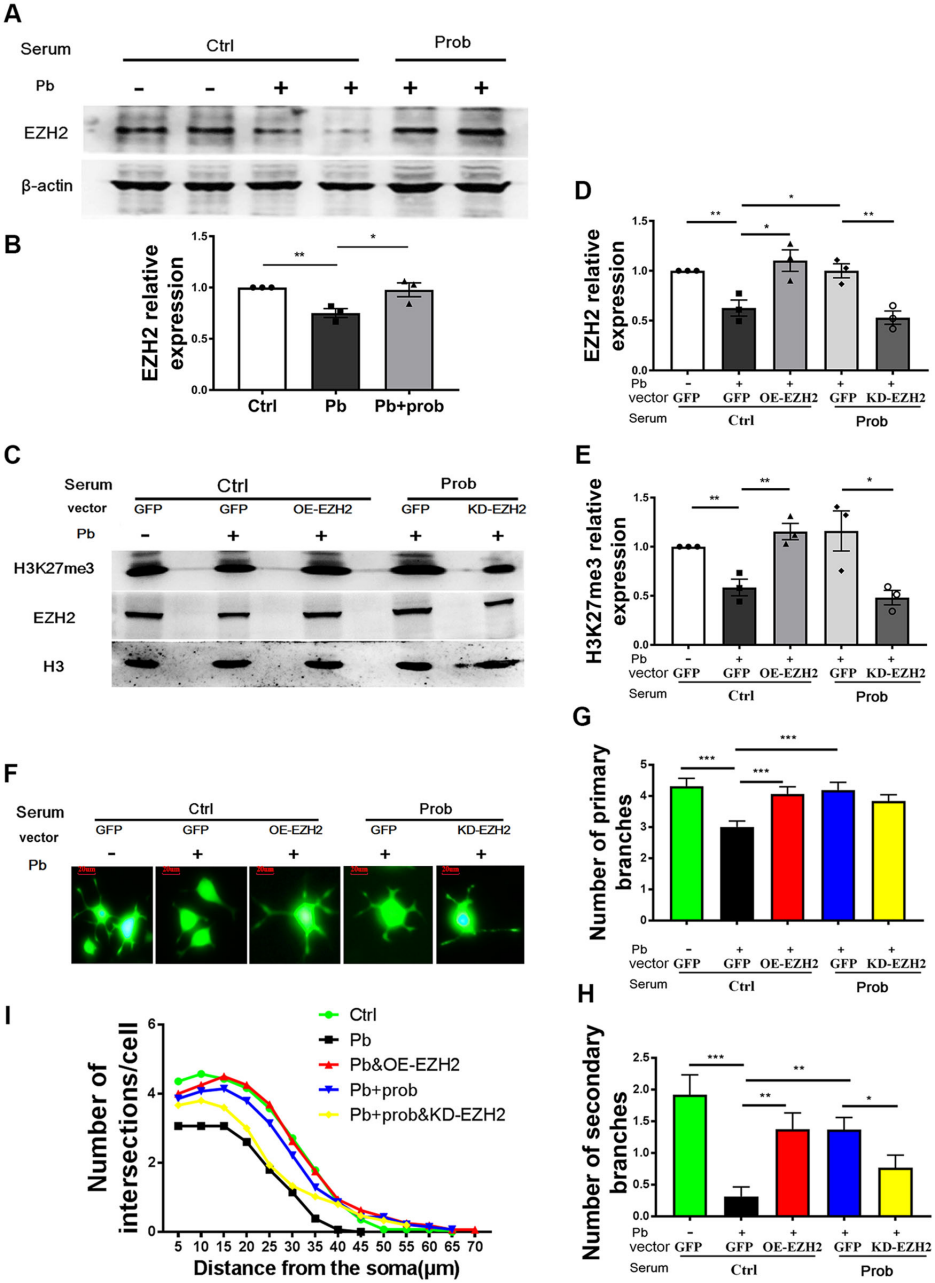
EZH2-H3K27me3 mediated the probiotic alleviation of lead neurotoxicity. Immunoblots (**a**) and quantification (**b**) of EZH2 levels in PC-12 cells supplemented with rat serum (*n* = 3). **c** Immunoblots of H3K27me3 and EZH2 levels in PC-12 cells transfected with EZH2-overepressing (OE-EZH2), EZH2-knockdown (KD-EZH2), or empty (GFP) plasmid (*n* = 3), and the quantifications of EZH2 (**d**) and H3K27me3 (**e**) are shown. **f** Representative images of neurite outgrowth in PC-12 cells upon various treatments; the scale bar represents 20 μm. Number of primary branches (**g**), secondary branches (**h**), and sholl analysis (**i**) of neurite outgrowth of PC-12 cells upon various treatments (~50 cells). Ctrl cells treated with serum of normal rats, prob cells treated with serum of probiotic rats, Pb cells treated with lead and normal rat serum, Pb+prob cells treated with lead and probiotic rat serum; the transfection of corresponding plasmids was involved in **b**–**f**. The data are represented as mean ± SEM; ***p < 0.001, **p < 0.01, *p < 0.05, *p* value was FDR corrected in multiple (>3) comparisons.

To sort out the causal–effect relationship between microbial normalization and neural epigenetic changes, blood serum of rats colonized with probiotics were collected and supplemented to the growth media of PC-12 cells, a model neural cell line. Cells were pretreated with lead to establish a neural damage model, and serum from untreated rats was used as control. The validity of the cellular model was demonstrated by measuring the expression level of EZH2, when the addition of probiotic serum prevented the decrease of EZH2 caused by lead exposure (*p* < 0.5 vs. pb group, Fig. 6a, b). One-way ANOVA revealed significant effect of treatment on the EZH2 levels (*F* (2, 6) = 8.722, *p* = 0.0168). In addition, with the transfection of OE-EZH2 or KD- EZH2 plasmids (Fig. 6c–e), the H3K27me3 level was accordingly changed in response to EZH2 intervention, showing a concerted action under the tested circumstances.

EZH2-H3K27me3 was then examined of their roles in bridging microbiota and neural function. Profiling neurite outgrowth of the induced PC-12 cells (Fig. 6f), it was discovered that overexpression of EZH2 improved the injured neurite growth induced by lead (FDR < 0.001 for primary branches; FDR < 0.01 for secondary branches, vs. pb+ctrl serum group, Fig. 6g, h), generating a parallel phenotype with probiotic serum. Moreover, as manifested by the secondary branches of outgrown neurites (FDR < 0.05, vs. pb+probiotic serum group, Fig. 6h) and sholl analysis (Fig. 6i), genetic inhibition of EZH2 led to a re-damage toward neurite growth, even though it had been previously rescued by probiotic serum. Together with the observation that the serum supplement increased the EZH2 level (Fig. 6a), a conclusion could be drawn: EZH2-H3K27me3 plays important roles in mediating the influence of microbial modulation on the lead neurotoxicity.

### IL-6 mediated EZH2 changes and the resulting memory recovery

**Fig. 7:**
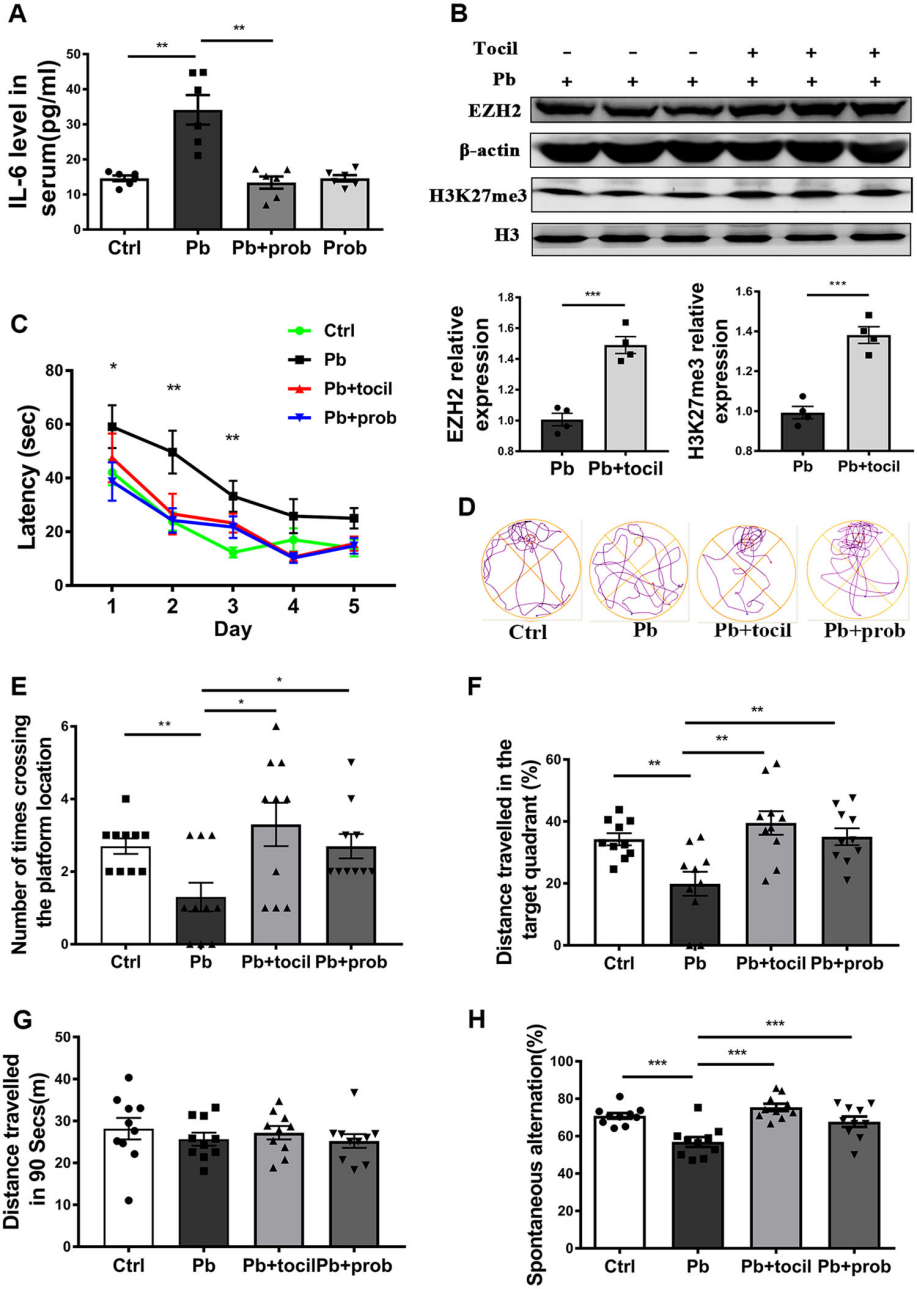
IL-6 mediated EZH2 changes and the resulting memory recovery. **a** The concentration of IL-6 in the serum of each group of rats, as detected by ELISA (*n* = 6). **b** Immunoblots and quantification of EZH2 and H3K27me3 levels in the hippocampus of adolescent rats, in response to tocilizumab injection (*n* = 4). **c**–**g** MWM tests assessing the capacities of rats to be trained to find the hidden platform (*n* = 10). Latency of rats to find the platform was recorded and analyzed during the training days (**c**). On the test day, based on their respective moving tracks (**d**), number of times crossing the hidden platform (**e**) and distance traveled in the target quadrant (**f**) were counted at PND 61. The total distance traveled was also recorded to evaluate the locomotor movement of the tested rats (**g**). **h** Y-maze test assessing the capacities of rats to perform the spontaneous alteration. Spontaneous alteration percentage of each group was shown (*n* = 10). Ctrl non-treated rats, Pb lead-treated rats, Pb+prob lead and probiotics-treated rats, Pb+tocil lead and tocilizumab-treated rats. The data are represented as mean ± SEM; ***p < 0.001, **p < 0.01, *p < 0.05. For **c**, two-way ANOVA was performed and asterisk (*) refers to the significance of differences between the Pb and Ctrl groups on the indicated training day.

Microbiome–gut–brain axis was thought to be regulated by the immune or alternative pathways^13,50^. Considering that rat blood serum was composed of multiple immunomodulatory factors, ELISA was performed to identify the immune factors responsible for the memory improvement. Among the pro- and anti-inflammatory factors under study, pro-inflammatory factor IL-6 was aberrantly stimulated or resumed with the treatment of lead (*p* < 0.01 vs. ctrl group) or lead+probiotics (*p* < 0.01 vs. lead group), respectively (Fig. 7a), while a typical anti-inflammatory factor IL-10 displayed an opposite changing propensity (Supplementary Fig. S4). Both (lead *F* (1, 20) = 15.09, *p* = 0.0009) and probiotics (*F* (1, 20) = 19.40, *p* = 0.0003) had significant effects on the IL-6 levels, and their interaction was significant (*F* (1, 20) = 19.39, *p* = 0.0003). Therefore, the lead-led peripheral inflammation was mitigated through probiotic supplementation. We subsequently obtained a fuller characterization of innate immune markers in the brain by measuring their mRNA levels in the studied settings by using primes listed in Supplementary Table S1. Consistent with serum levels, lead exposure increased the expression of IL-6 in the rat hippocampus, which was restored by probiotic supplementation. Other negatively responsive immune factors might include C-X-C chemokine motif ligand 1 and G-CSF (Supplementary Fig. S5). Interestingly, no apparent expression of IL-10 was detected in the studied cerebral context (data not shown).

Tocilizumab is a specific antagonist against IL-6 receptor, and it was thus injected in vivo to investigate the roles of IL-6 in the studied gut–brain connections. According to Fig. 7b, the use of tocilizumab increased the level of hippocampal EZH2 and H3K27me3 compared to the lead-treated rats (*p* < 0.001). This observation is consistent with cases of probiotic intervention (Fig. 6a–e), suggesting that the changes of EZH2 and H3K27me3 in the studied context were related to the altered level of IL-6.

Concerning the spatial memory characterized by MWM test, the durations of lead-exposed rats to find the platform was shortened upon the additional treatment of tocilizumab during training sessions (*p* < 0.05 for Pb+tocil vs. Pb, Fig. 7c). Repeated-measures ANOVA showed that both the training days (*F* (3.052, 97.66) = 23.52, *p* < 0.001) and treatment (*F* (3, 32) = 11.51, *p* < 0.001) had main effect on the behavioral outcome, and their interaction was insignificant (*F* (12, 128) = 0.6645, *p* = 0.7825). On the test day, the additional tocilizumab-treated rats performed better in locating the target platform than the lead-treated rats, as evidenced by crossing times (*p* < 0.05, Fig. 7d, e) and distance traveled in the target quadrant (*p* < 0.05, Fig. 7d, f). With the comparable rescue effect with probiotics (*p* > 0.05 for Pb+tocil vs. Pb+prob) (Fig. 7e, f), implications of IL-6 in the studied gut–brain communication might be suggested. Besides, no differences were observed in the total movement among the tested groups (Fig. 7g). In addition, according to the results of Y-maze test, the lead-led damage on working memories could also be alleviated by tocilizumab treatment (*p* < 0.001 vs. pb), an effect comparable with probiotic administration (Fig. 7h). The Y-maze test supports the mediatory roles of IL-6 in connecting microbial orientation with the studied brain behavior.

## Discussion

Chelation therapy was ineffective in treating low-level lead exposure and failed to reverse the associated memory deficits^51^. Another drawback is difficulties in addressing the spatiotemporal separation of adverse and intervention courses^9^. Microbe-based therapy is otherwise an alternative approach to address the issue. The long-range crosstalk between GM and brain underlies the ability of this therapy to treat symptoms of memory deficits. Based on its GRAS (generally regarded as safe) status, the use of probiotics was a safe and well-tolerated means from the heath perspective. In addition, this strategy could be potentially expanded to any customized approaches at restructuring GM toward a specific situation of brain dysfunction. Nonetheless, it should not be ignored that the neuromodulatory effect of probiotic strains is contradictive in some cases. For instance, the use of the prototype “psychobiotic,” namely, *Lactobacillus rhamnosus* JB-1, failed to modulate cognitive performance in male subjects^52^. Contrastingly, treatment with multispecies probiotics reduced depressive symptoms in depressed patients compared to placebo^53^. Consequently, someone argues that microbes with exclusive diversity are frequently not sufficient to induce a comprehensive phenotypic advantage^52^. The effect of multispecies probiotics used in this study supported this argument.

Intricate relationships exist between the intestinal bacterial community and the CNS. In one instance, the disrupted microbiome was associated with the development of autism spectrum disorder (ASD)-related defects^16^. In another case, injection of *Lactobacillus rhamnosus* JB-1 improved the emotional behavior of mouse^54^. These evidences supported the notion that impact of GM on brain behavior was dependent on the genetic and physiological context and specific strains involved. Although the adverse effect of lead on spatial memory is extensively verified, this neurotoxicity was not previously antagonized by the probiotic treatment, wherein the mixed use of *Bifidobacterium* and *Lactobacillus* strains showed a rescue effect in the present study (Figs. 1 and 2). The successful intervention unveiled the considerable plasticity of GM. This “super-organ” might be subjected to variable environmental cues and oriented manipulations.

Firmicutes and Bacteroidetes are the predominant bacteria inhabiting the human gastrointestinal tract^55^, enabling their relative abundance a common indicator of the overall microbial architecture. A higher ratio of F/B was usually implicated in the brain dysfunction or metabolic abnormalities^20,56^. Interestingly, lead-led memory damage was also accompanied by a higher F/B ratio (Fig. 3c, d), which was partially normalized by the introduction of probiotic bacteria. In light of it, F/B rebalancing was supposed to be the key microbiota event in the alleviation of lead-led memory impairment. The F/B alterations were consistent with a recent discovery studying lead exposure and body weight increases^57^. Nonetheless, conflicting results occurred when *Lactobacillus* and some individual species were taken into account in response to lead exposure. These discrepancies might result from the genetic variances, distinct exposure dosage/period, and sex differences. Overall, the abnormal F/B ratio, as well as other characteristic taxa of GM, did contribute to multiple adverse outcomes of chronic lead exposure, which was subsequently reinstated with probiotic interference.

The interactions among microbial species resulted in a highly complex network of inter-related signals, making it challenging to establish the cause and effect relations between microbial dynamics and a given phenotype^58^. We attempted to use a neuronal cellular model to investigate the routes linking gut–brain functions. To address the trans-systematic difficulties, animal trials were integrated to supply the neuronal cells with the active substances they were supposed to encounter in vivo. The use of model mimicked the rescue effect of probiotics (Fig. 6a), an evidence that it could, at least partially, reflect the bona fide trans-systematic signal originating from microbial changes. Another strength of this model is the availability of genetic manipulations, which might favor the evaluation of neural epigenetic roles in the studied gut–brain crosstalk. The limitation of this model includes the infidelity to mimic a genuine cellular environment by the use of a neural cell line. Still, this experiment gave a new perspective for mechanistic studies involved in trans-systematic process.

It seems plausible to explain gut–brain crosstalk in the perspective of cerebral gene expression. However, there was a paucity of direct evidence for the role of neural epigenetics in remodeling host–microbiota interactions^59^. Microbial colonization was recently found to regulate global histone acetylation and methylation in multiple host tissues^60^. And particularly, Alenghat et al. discovered that HDAC3 was essential in maintaining commensal bacteria-dependent intestinal homeostasis^61^. We extended the scope of epigenetic function to the microbiome–gut–brain axis, highlighting H3K27me3 and its cognate methyltransferase, EZH2. As a global regulator, it could be speculated that alterations of total H3K27me3 level in hippocampus should lead to a genome-wide redistribution of this methylated form of histone, as well as a consequent reprogramming of various functional genes. Profiling the entire accessibility landscapes of H3K27me3 in response to lead and probiotic treatment will aid in deciphering the detailed molecular aspects, which needs to be explored in further investigations.

What factors affected the existing status of EZH2-H3K27me3? Inflammation pathway is regarded as the competent candidate, based on its implications in gut–brain communication. Prebiotic administration led to a normalized IL-6 level^13^, and a similar effect was found in the application of probiotics to antagonize lead neurotoxicity (Fig. 7a). IL-6 was identified as a sensible effector and mediator among the various immunological factors. Consistent with our findings, associations of IL-6 with the development of ASD, bipolar disorders, or other psychiatric diseases have long been appreciated^62^. The advantages of IL-6 as a gut–brain messenger might be related to the convenience of the immune-to-brain pathways, which consisted of the convergent action of neural activation and cytokine propagation^63^.

According to findings here, a hypothesis could be proposed: in the context of microbiome–gut–brain communications, probiotics interfere with the intoxicating pathway of lead, namely, gut dysbiosis–IL-6 stimulation–H3K27me3 abnormality, through which it partially prevented the memory dysfunction. The probiotic antagonism derives from its capacity to modulate GM and/or converges on the hippocampal H3K27me3 balance. In summary, the GM–IL-6–H3K27me3 pathway provided an alternative route to explain the probiotic antagonism against lead neurotoxicity (Supplementary Fig. S6). This integrated link could be substantiated by the IL-6 intervention trials (Fig. 7). In this trial, it was found that probiotic treatment could simultaneously resume the serum IL-6 level, as well as the H3K27me3 level in the brains and their cognitive behavior, suggesting that probiotic treatment be regarded as the common causes of the serial response. In addition, IL-6 blockade led to a beneficial epigenetic and behavioral outcome in the rat brains, establishing a cause–effect link in the immune brain. Owing to the crosstalk among the studied roles, microbes–immune–brain link was established. Still, the effect spectrum of this pathway in other distinct gut–brain conditions awaits to be further defined.

In conclusion, lead-led memory dysfunction, a worldwide health concern, was alleviated through long-term probiotic intervention. The disrupted composition of GM was reshaped and partially normalized by the probiotic treatment. Subsequently, the levels of EZH2-H3K27me3 changed concomitantly with the microbial alterations and the corresponding memory status. In the end, the cytokine IL-6 contributed to the alterations of EZH2-H3K27me3, as well as neurotoxic phenotypes. This study expanded the mechanisms underlying interactions between GM and neurotoxicity to the layer of cerebral epigenetics. This study might shed new light on the studies pertaining to the pathogenesis and therapeutics of neurodegenerative diseases with environmental cues.

## Supplementary information

Supplementary Table S1

Supplementary Figure S1

Supplementary Figure S2

Supplementary Figure S3

Supplemetary Figure S4

Supplementary Figure S5

Supplemetary Figure S6

Supplementary Video S1

Supplementary Video S2

Supplementary Video S3
