# Supplementary material for: Long-term probiotic intervention mitigates memory dysfunction through a novel H3K27me3-based mechanism in lead-exposed rats
Source: Transl Psychiatry. 2020 Jan 22;10:25. doi: 10.1038/s41398-020-0719-8 (PMC7026181; doi:10.1038/s41398-020-0719-8)
Supplement: Supplementary file 1 — Supplementary Table S1 [file 41398_2020_719_MOESM1_ESM.docx]

**Supplementary Table S1. primers used in this study**

| **Primers** | **Sequences (5’-3’)** | **Gene** |
| --- | --- | --- |
| TNF-α57F | TCCCAGAAAAGCAAGCAACCAG | TNF-α |
| TNF-α305R | CCGAAGTTCAGTAGACAGAAGAGCG |  |
| IL-6 477F | AAGCCAGAGTCATTCAGAGCAA | IL-6 |
| IL-6 635R | GGATGGTCTTGGTCCTTAGCC |  |
| IFN-γ 377F | ACAACCCACAGATCCAGCACAA | IFN-γ |
| IFN-γ 482R | AATCAGCACCGACTCCTTTTCC |  |
| IGF-1 91F | ACTCTGCTTGCTCACCTTTACCA | IGF-1 |
| IGF-1 263R | CATCCACAATGCCCGTCTG |  |
| GCSF 805F | TGGAGGGCAGGGAAGGAGATA | GCSF |
| GCSF 1063R | CGGGGTCAGGAAAACCTACAAC |  |
| IL-1b 54F | GCTATGGCAACTGTCCCTGAAC | IL-1β |
| IL-1b 225R | CGAGATGCTGCTGTGAGATTTG |  |
| Cxcl1 1230F | TGAACGCTGGCTTCTGACAA | CXCL1 |
| Cxcl1 1512R | CTCATCTCTCCGCCCTTCTTC |  |
| ActinF | CCTGAAGTACCCCATTGAAC | β-actin |
| ActinR | GAGGTCTTTACGGATGTCAAC |  |
