# Supplementary figures and images for: Long-term probiotic intervention mitigates memory dysfunction through a novel H3K27me3-based mechanism in lead-exposed rats
Source: Transl Psychiatry. 2020 Jan 22;10:25. doi: 10.1038/s41398-020-0719-8 (PMC7026181; doi:10.1038/s41398-020-0719-8)

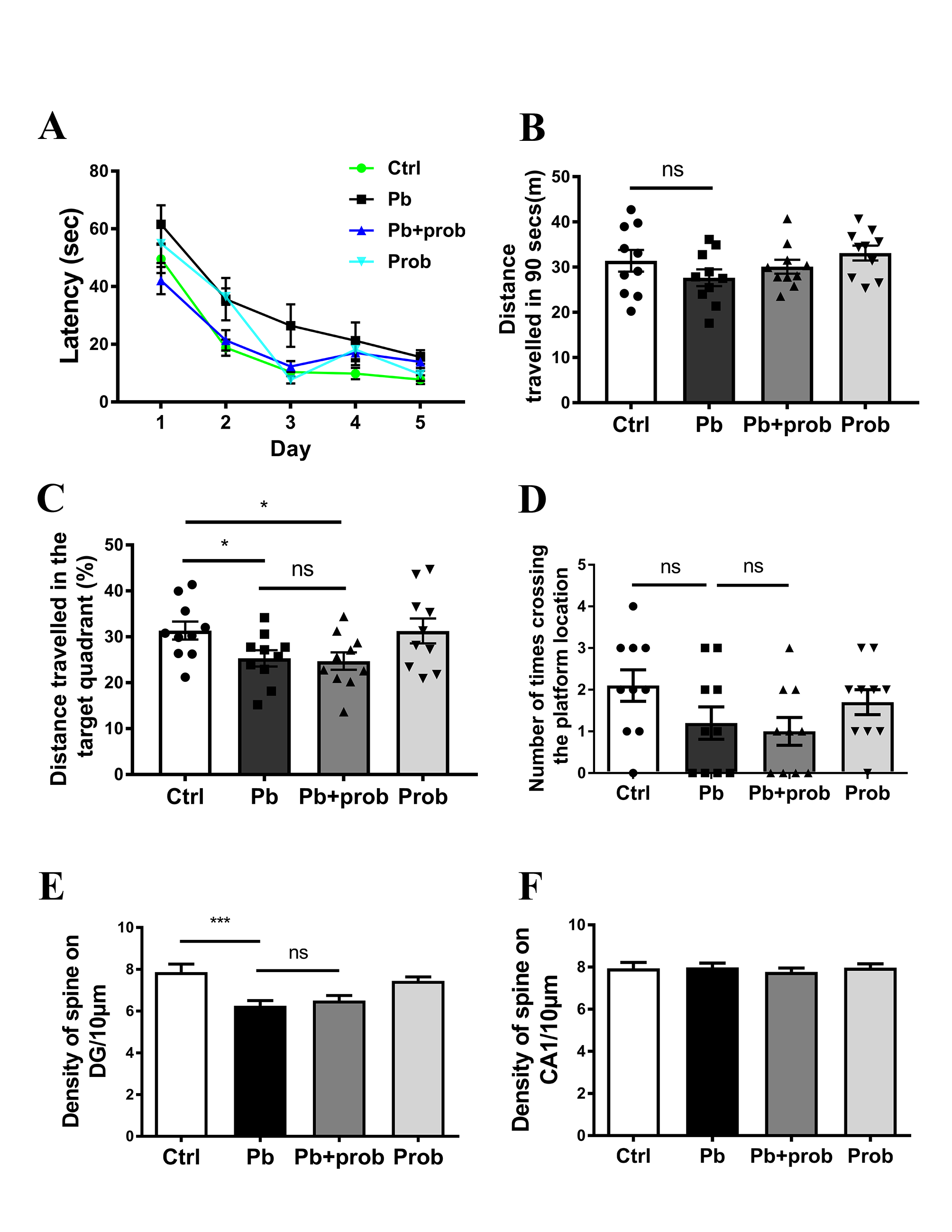

Supplement: Supplementary file 2 — Supplementary Figure S1 [file 41398_2020_719_MOESM2_ESM.tif]

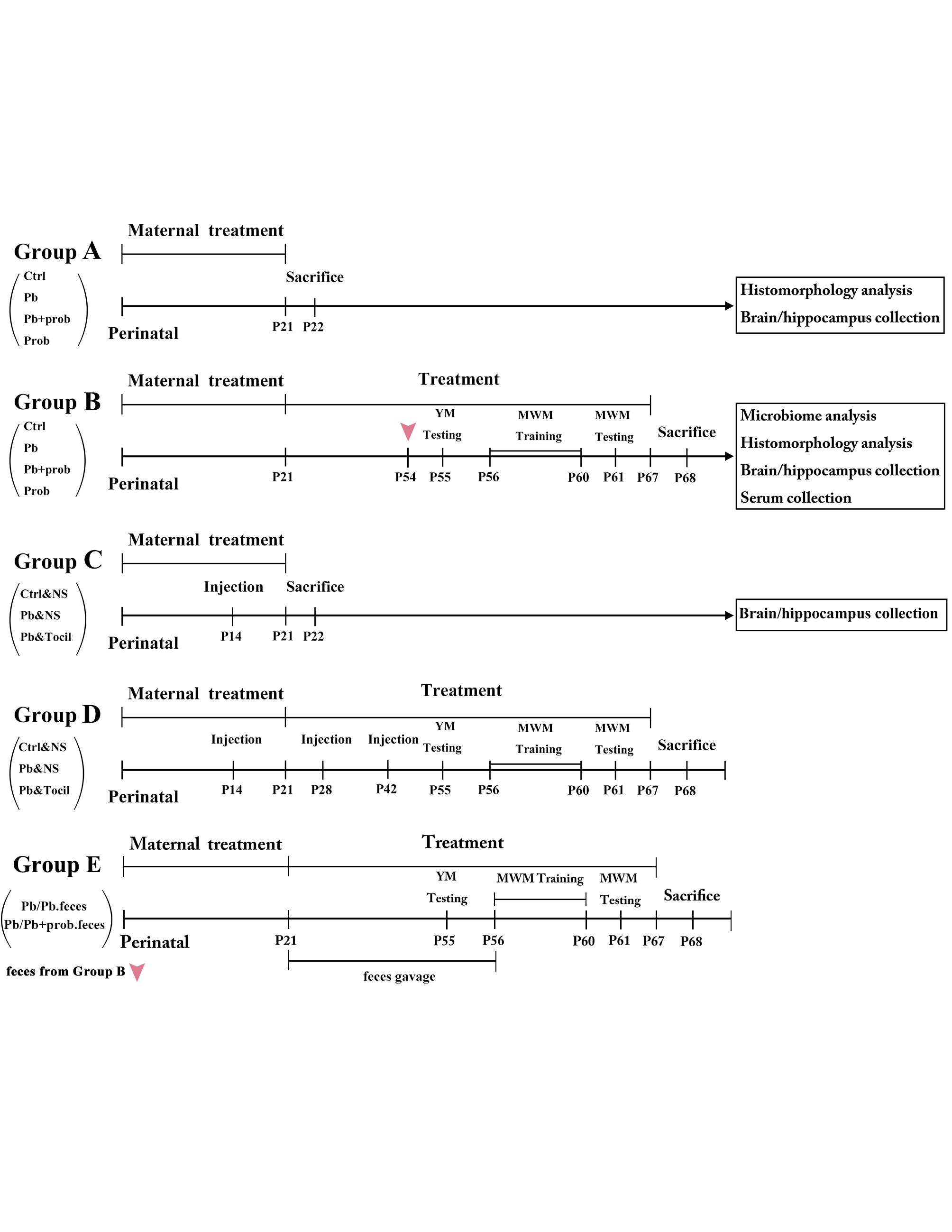

Supplement: Supplementary file 3 — Supplementary Figure S2 [file 41398_2020_719_MOESM3_ESM.tif]

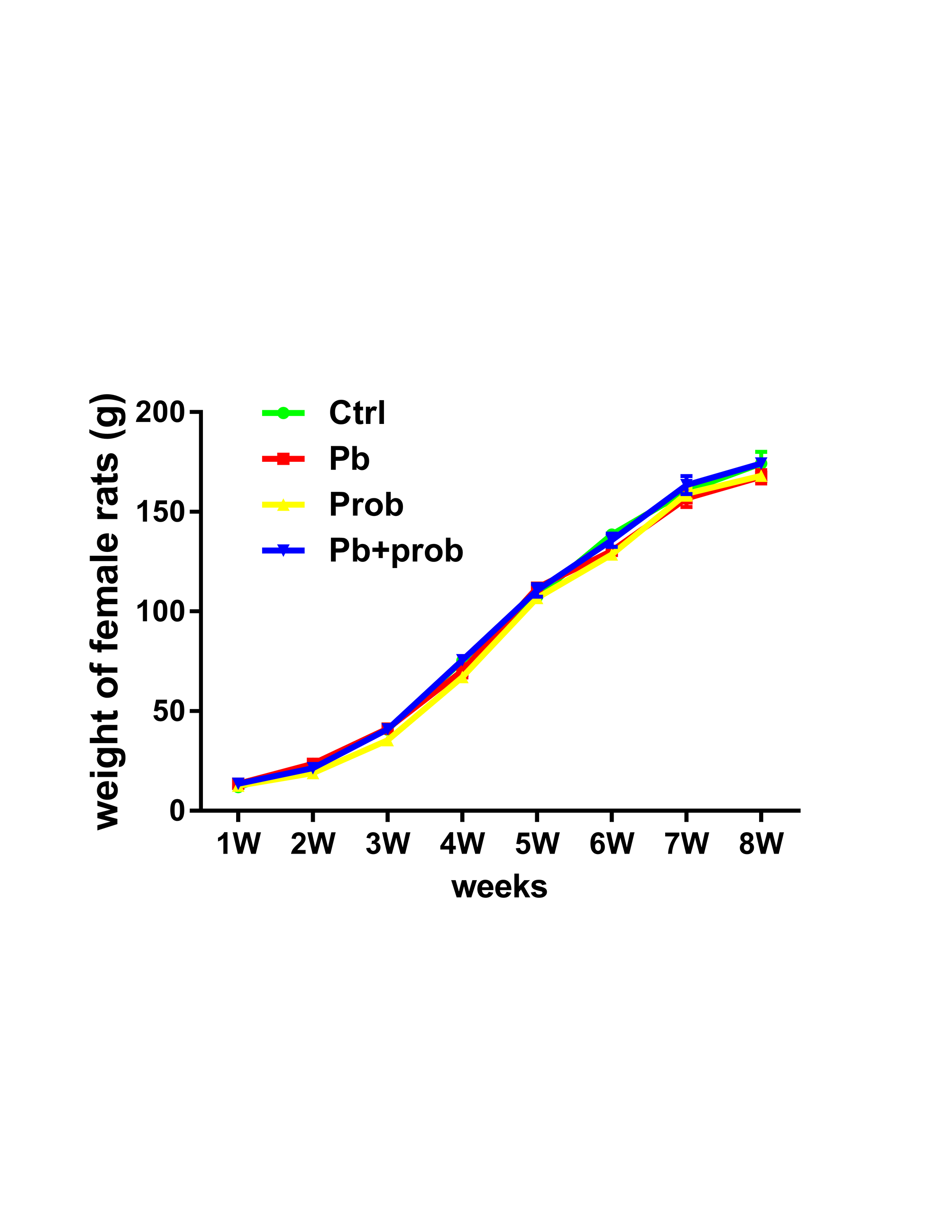

Supplement: Supplementary file 4 — Supplementary Figure S3 [file 41398_2020_719_MOESM4_ESM.tif]

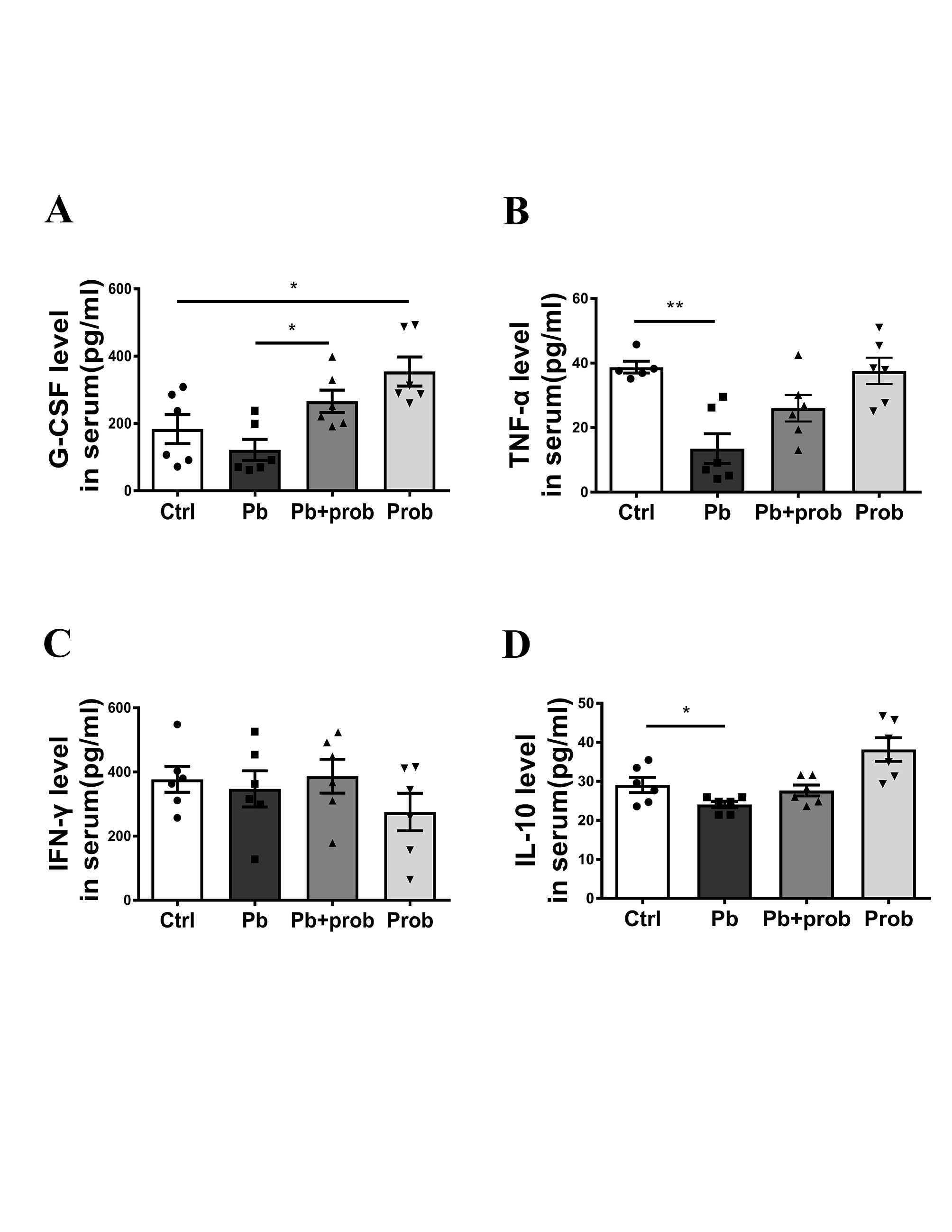

Supplement: Supplementary file 5 — Supplemetary Figure S4 [file 41398_2020_719_MOESM5_ESM.tif]

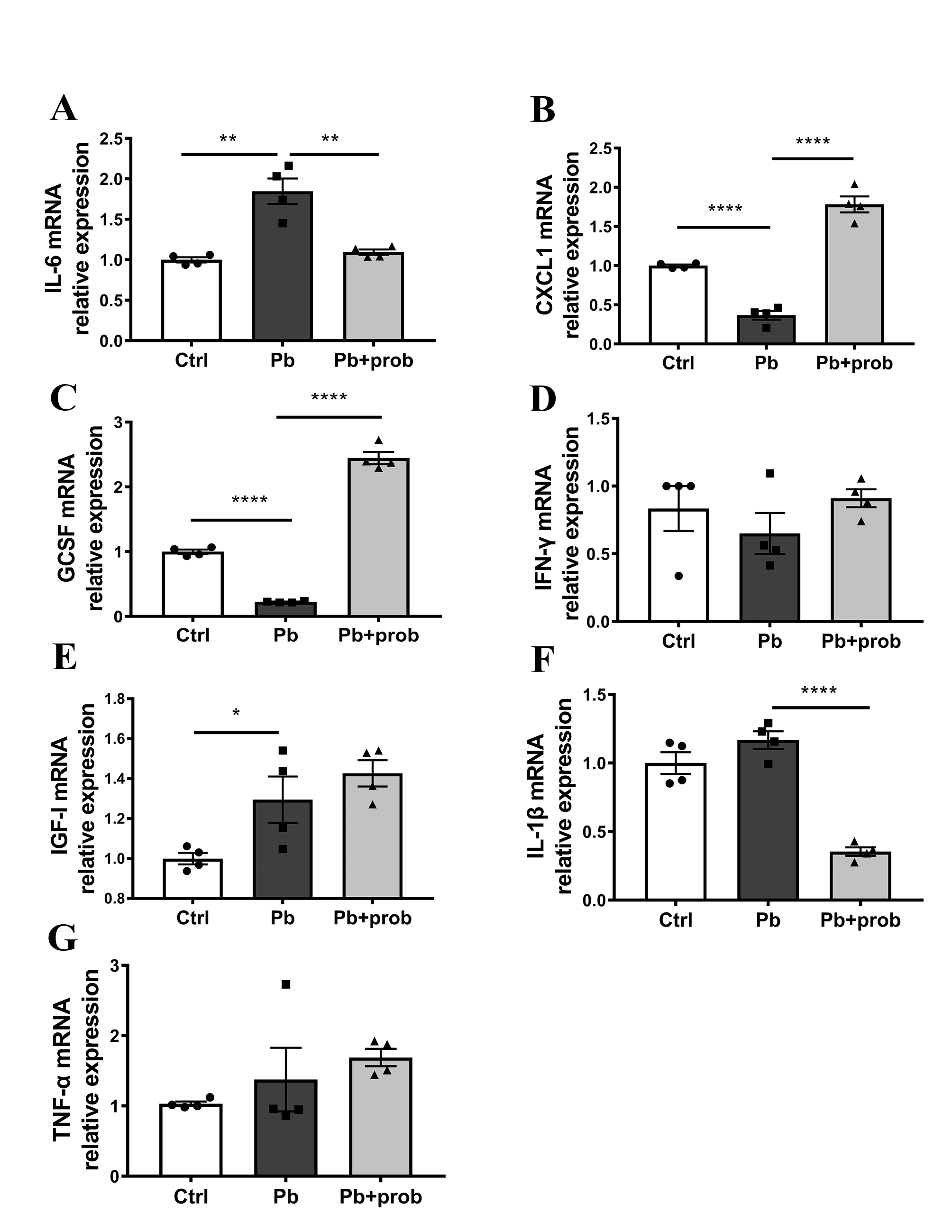

Supplement: Supplementary file 6 — Supplementary Figure S5 [file 41398_2020_719_MOESM6_ESM.tif]

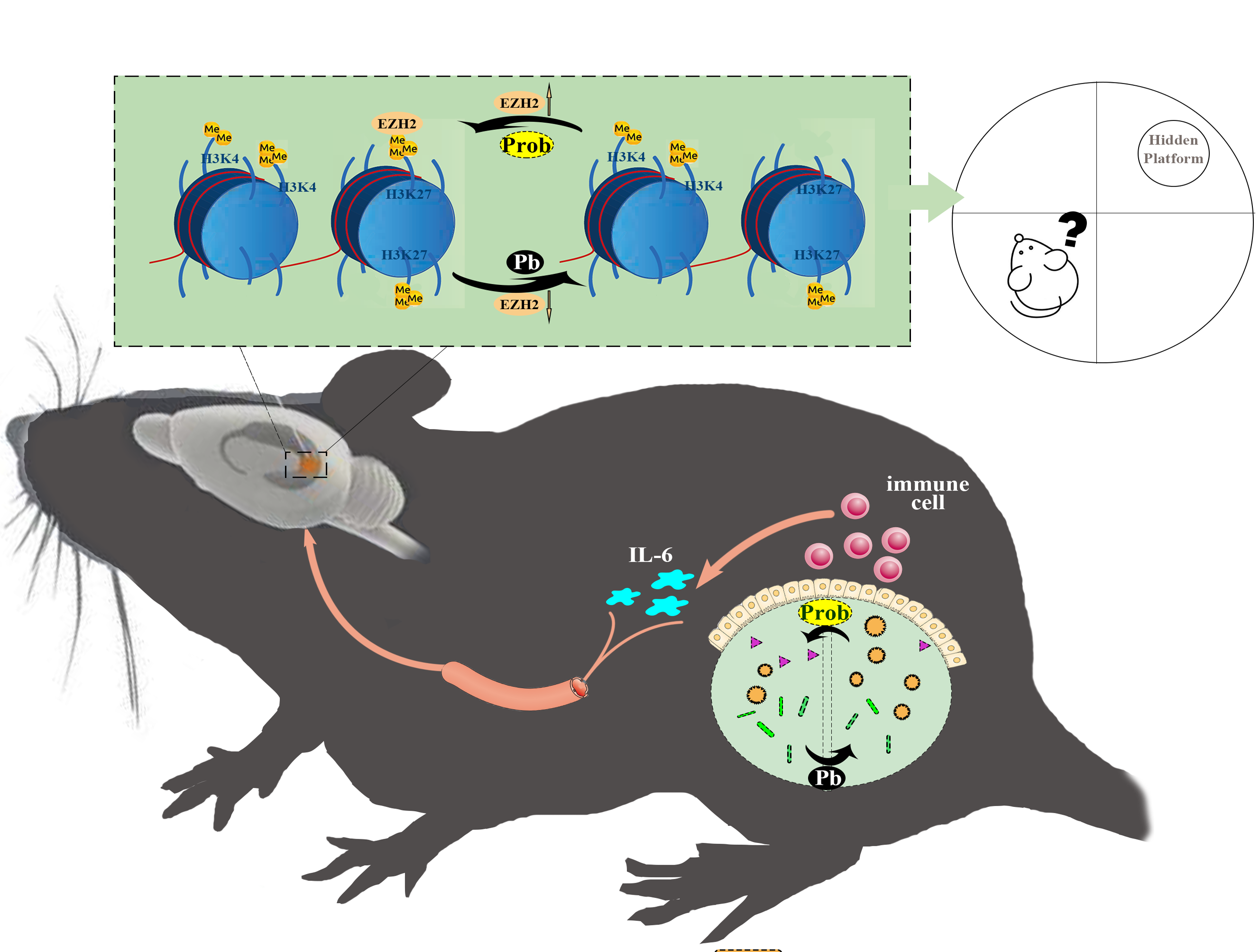

Supplement: Supplementary file 7 — Supplemetary Figure S6 [file 41398_2020_719_MOESM7_ESM.tif]
